# Supplementary material for: Hypoxia-induced proteasomal degradation of DBC1 by SIAH2 in breast cancer progression
Source: eLife. 2022 Aug 1;11:e81247. doi: 10.7554/eLife.81247 (PMC9377797; doi:10.7554/eLife.81247)
Supplement: Supplementary file 1. — List of representative proteins identified by SIAH2 Co-IP/MS and the number of peptides for each protein-identified peptide are indicated. [file elife-81247-supp1.docx]

Supplementary Table 1: Identification of DBC1 as a SIAH2-associated protein.

| Protein | Number of peptides |
| --- | --- |
| 2-oxoglutarate dehydrogenase | 35 |
| 2-oxoglutarate dehydrogenase-like | 16 |
| Serine/threonine-protein kinase LATS2 | 5 |
| Cell cycle and apoptosis regulator protein 2 (CCAR2) | 4 |
| Nuclear respiratory factor 1 (NRF1) | 4 |
| SH3 domain-containing RING finger protein 3 (POSH2) | 4 |
