# Supplementary material for: Hypoxia-induced proteasomal degradation of DBC1 by SIAH2 in breast cancer progression
Source: eLife. 2022 Aug 1;11:e81247. doi: 10.7554/eLife.81247 (PMC9377797; doi:10.7554/eLife.81247)
Supplement: Supplementary file 2. — The relationship between the clinicopathological characteristics of breast cancer patients and the expression level of DBC1. [file elife-81247-supp2.docx]

Supplementary Table 2 Patient characteristics based on DBC1 expression

|  | DBC1 expression | | | | |
| --- | --- | --- | --- | --- | --- |
| Variables | Total | | Negative/+ | ++/+++ | *p-value* |
| Age |  | |  |  | 0.89037 |
| >50 years | 26 | | 14 (53.8%) | 12 (46.2%) |  |
| ≤ 50 years | 74 | | 41 (55.4%) | 33 (44.6%) |  |
| T stage |  | |  |  | 0.01971 |
| T1/T2 | 73 | | 35 (47.9%) | 38 (52.1%) |  |
| T3/T4 | 27 | | 20 (74.1%) | 7 (25.9%) |  |
| AJCC stage |  | |  |  | 0.00027 |
| I/IIa | 51 | | 19 (37.3%) | 32 (62.7%) |  |
| IIb/III | 49 | | 36 (77.6%) | 13 (22.4%) |  |
| Lymph node metastasis | |  |  |  | 0.24095 |
| N0 | 64 | | 38 (59.4%) | 26 (40.6%) |  |
| N1/2 | 36 | | 17 (47.2%) | 19 (52.8%) |  |
| Ki67 positive |  | |  |  | 0.03145 |
| ≤ 20% | 69 | | 33 (47.8%) | 36 (52.2%) |  |
| >20% | 31 | | 22 (71.0%) | 9 (29.0%) |  |
| PR |  | |  |  | 0.23773 |
| Negative | 58 | | 29 (50.0%) | 29 (50.0%) |  |
| Positive | 38 | | 24 (63.2%) | 14 (36.8%) |  |
| Her-2 |  | |  |  | 0.37531 |
| Negative | 51 | | 26 (51.0%) | 25 (49.0%) |  |
| Positive | 45 | | 27 (60.0%) | 18 (40.0%) |  |
| ER |  | |  |  | 0.44828 |
| Negative | 45 | | 23 (51.1%) | 22 (48.8%) |  |
| Positive | 51 | | 30 (58.8%) | 21 (41.2%) |  |
